# Natural Progression of Rheumatic Aortic Valve Disease Following Mitral Valve Intervention: A 16-Year Single-Center Experience

**DOI:** 10.1155/crp/6689214

**Published:** 2025-06-17

**Authors:** Seok Hyun Kim, Ji Hoon Lim, Sang Hyun Lee, Mi Hee Lim, Chee-Hoon Lee, Min Ho Ju, Hyung Gon Je, Yong Hyun Park

**Affiliations:** ^1^Department of Internal Medicine and Research Institute for Convergence of Biomedical Science and Technology, Division of Cardiology, Pusan National University Yangsan Hospital, Pusan National University School of Medicine, Yangsan, Republic of Korea; ^2^Department of Cardiovascular Surgery and Research Institute for Convergence of Biomedical Science and Technology, Pusan National University Yangsan Hospital, Pusan National University School of Medicine, Yangsan, Republic of Korea

**Keywords:** aortic regurgitation, aortic stenosis, mitral regurgitation, mitral stenosis, rheumatic heart disease

## Abstract

**Background:** Recognizing the natural progression of the remaining valve disease following the intervention of a single valve is crucial in multiple rheumatic valvular diseases as often encountered in clinical practice. We aimed to investigate whether performing mitral valve (MV) intervention alone for multiple rheumatic MV and aortic valve (AV) disease is safe.

**Hypothesis:** Rheumatic AV disease progresses slowly and severity does not differ significantly after MV intervention.

**Methods:** We retrospectively investigated the progression of AV disease with rheumatic changes following MV intervention in a single tertiary center. Among 890 patients initially screened, 76 patients met the criteria for assessment.

**Results:** Six patients fell under severe aortic stenosis (AS) definition-wisely and four of them were classified as low-flow low-gradient severe AS despite normal ejection fraction. Eventually, four patients were found to have true-severe AS at a median follow-up period of four years (mean 5.8 years) and only one of them underwent AV surgery for severe AS per se. None of the patients with aortic regurgitation deteriorated to severe.

**Conclusions:** Only a small portion of rheumatic AV involvement progresses to severe AS after MV intervention, and performing MV intervention for severe mitral stenosis or mitral regurgitation in patients with concurrent mild or moderate AS or aortic regurgitation due to rheumatic changes is reasonable.

## 1. Introduction

Clinicians frequently encounter cases of multiple valvular heart disease, wherein the combination of stenotic or regurgitant lesions occurs on separate cardiac valves [1]. Rheumatic etiologies constitute a significant portion of cardiac valve disease, with the potential to affect either the mitral valve (MV) or aortic valve (AV), leading to the development of multiple valve disease. Rheumatic valve disease is a chronic sequel of acute rheumatic fever, following streptococcal pharyngotonsillitis, and it progresses slowly. The MV is most frequently affected, and the AV and tricuspid valve (TV) may be directly implicated when MV involvement is present [2]. Rheumatic cardiac valve disease, which disproportionately affects low socioeconomic communities, is responsible for up to 1.4 million annual deaths worldwide. Despite its clinical relevance, it has historically received less attention, but recent years have witnessed a surge of interest in this condition [3, 4]. Even in industrialized countries, rheumatic heart disease remains a pertinent issue. A recent cross-sectional study conducted in Korea on cardiac valve disease revealed that rheumatic etiology predominates in mitral stenosis (MS) (74.6%), and accounts for 11.6% of primary mitral regurgitation (MR), 9.1% of aortic regurgitation (AR), and 7.7% of aortic stenosis (AS) cases [5]. Especially in cases of rheumatic multiple valve disease, intervening in a single valve often leads to a change in the severity of the remaining valve disease, rectifying any overestimations or underestimations due to interlesion interactions [6]. As such, the clinical decision-making process regarding which valve to intervene upon is of paramount importance. Thus, recognizing the natural progression of the remaining valve disease following the intervention of a single valve is crucial. Unfortunately, there is a scarcity of literature on the natural progression of rheumatic AV disease after MV intervention [7, 8]. Therefore, the aim was to assess the progress of nonintervened AV disease in patients with multivalvular chronic rheumatic heart disease, undergoing MV intervention and also investigate the safety of intervening on MV alone, in the presence of less than severe AV disease.

## 2. Methods

Patients who demonstrated rheumatic involvement of the AV following MV intervention for rheumatic MS or MR, which encompassed MV replacement (MVR), percutaneous mitral balloon valvuloplasty (PMBV), and any other forms of MV intervention such as ringed annuloplasty at Pusan National University Yangsan Hospital between December 1st, 2008 and December 31st, 2023, were included. For patients who underwent multiple MV interventions, we considered the first intervention as the criteria for data collection. Three patients who had their initial MV intervention at another hospital were also included. Those who underwent concurrent or bygone intervention on the AV or lacked echocardiographic follow-up exceeding one year were excluded.

Two clinicians specializing in echocardiography determined whether the AV disease had a rheumatic etiology. The severity grading of cardiac valve disease adhered to current echocardiography guidelines [9–11].

In cases of AS, severity was assessed based on echocardiographic measurements, including maximal transaortic velocity (*V*_max_), mean transaortic pressure gradient (mPG), and aortic valvular orifice area (AVA), calculated using the continuity equation as the Doppler-derived effective orifice area. Echocardiography was performed using Vivid E90 (GE Healthcare, Illinois Chicago, U.S.A.), Vivid E9 (GE Healthcare, Illinois Chicago, U.S.A.), iE33 xMATRIX (Philips, Amsterdam, Kingdom of the Netherlands), and Acuson SC2000 (Siemens Healthineers, Erlangen, Germany). Unmeasured variables were retrospectively calculated when possible using TomTec Arena (TOMTEC, Unterschleißheim, Germany) during this investigation.

Defining severe AS remains somewhat elusive. In this study, we considered a case to be presumed severe AS if it met at least one of the three classical criteria: *V*_max_ > 4.0 m/s, mPG > 40 mmHg, or AVA < 1.0 cm^2^. High-gradient severe AS was defined as cases where Vmax > 4.0 m/s or mPG > 40 mmHg was met, with no significant reversible causes that could lead to overestimation of severity due to increased flow. Low-flow, low-gradient (LFLG) presumed severe AS (cases where AVA < 1.0 cm^2^ and mPG < 40 mmHg, with stroke volume index [SVi] < 35 mL/m^2^) required further classification. Assessing severity in these cases is particularly challenging, especially in patients with LFLG severe AS despite preserved ejection fraction (EF). In our study, we introduced the velocity-time integral (VTI) ratio as an additional hemodynamic criterion, based on the principle that parameters leading to pathological events in valvular heart disease should define true-severe AS. Using this VTI ratio, we were able to distinguish true-severe AS from pseudosevere AS within the LFLG subgroup.

The study protocol conforms to the ethical guidelines of the 1975 Declaration of Helsinki [12]. This retrospective cohort study received approval from the Pusan National University Institutional Review Board, with a waiver of informed consent (IRB number: 55-2024-052).

Statistical analyses were performed using R (Version 4.3.1, R Foundation for Statistical Computing, Vienna, Austria).

## 3. Results

**Figure 1:**
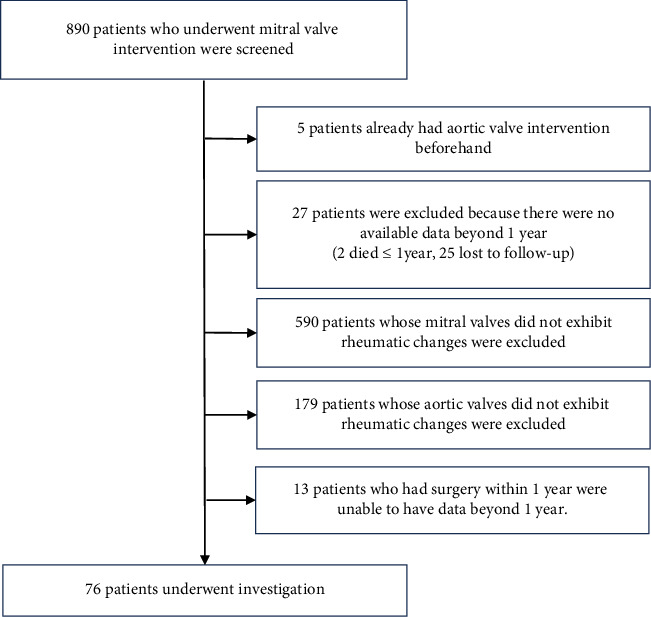
Patient sorting diagram. Patients who demonstrated rheumatic involvement of the AV following MV intervention for rheumatic MS or MR at Pusan National University Yangsan Hospital between December 1st, 2008 and December 31st, 2023, were included. Among 890 patients initially screened, 76 patients met the criteria for assessment.

A total of 890 patients who underwent MV intervention for rheumatic MS or MR without concurrent AV intervention were initially screened for eligibility. Among them, 76 patients had AV involvement of rheumatic etiology and fulfilled the inclusion criteria of our study (Figure 1). The baseline characteristics of these patients are outlined in Table 1. The mean age of the study population was 56.5 ± 9.6 years, with a female-to-male ratio of more than 2:1. Atrial fibrillation (AF) was observed in 60 patients. The median follow-up period was 4 years (mean 5.8 years, ranging from 1 to 31 years). MS was the most common indication for mitral intervention. None of the patients were prescribed long-term antirheumatic antibiotics.

### 3.1. Surgery

Mechanical MV was applied to 54 patients without any complications, except for one case that experienced unexpected cardiac arrest with successful resuscitation within a month of the surgery. Nine patients received a bioprosthetic MVR. Among them, one patient required a redo-MVR due to a tissue valve tear, while another patient underwent redo-MVR along with AV replacement (AVR) due to infective endocarditis. Ringed mitral annuloplasty was performed for one patient with severe MR.

### 3.2. PMBV

Out of the 12 cases who underwent PMBV, one underwent PMBV twice before requiring MVR, one case (Patient A, detailed below) underwent double valve replacement (DVR) for both the AV and MV after two PMBV procedures, and one case underwent MVR along with coronary artery bypass graft surgery nine years after PMBV due to unstable angina.

### 3.3. Progression of Rheumatic AS

**Figure 2:**
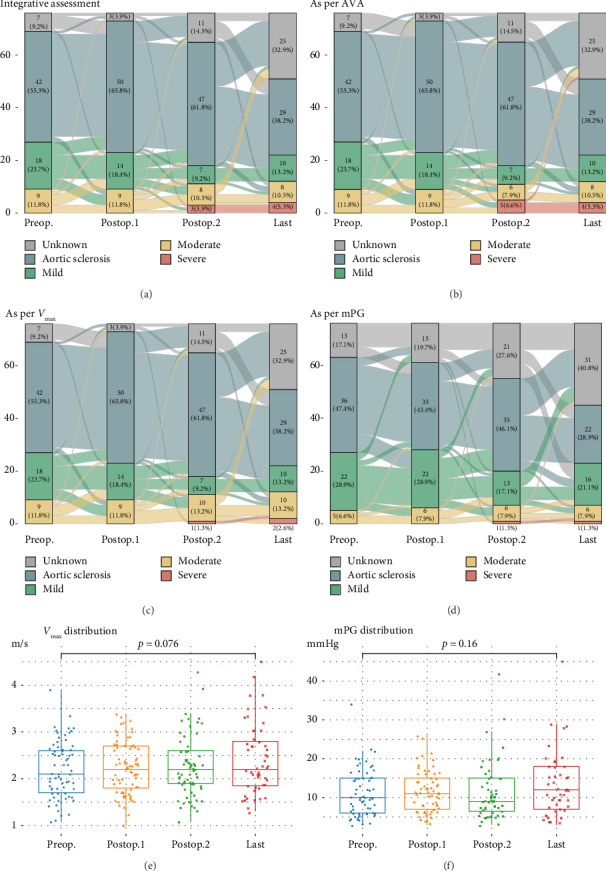
The changes in the grading of AS and distribution of individual *V*_max_ and mPG values. The changes in the grading of aortic stenosis as per four standards are presented. For each assessment standard, results with missing data across the four time points (*n* = 76) are given. Severe AS was rarely observed in the echocardiographic assessment immediately after surgery (Postop.1) but was most commonly identified at Postop.2. Beyond this point, further progression was uncommon, with minimal changes in AS severity observed in the echocardiographic data at the 5-year follow-up. (a) AS grading as per integrative assessment, incorporating the velocity-time integral ratio to classify low-flow, low-gradient severe AS despite preserved left ventricular ejection fraction. (b) AS grading as per AVA perspective, where AVA was calculated using the continuity equation as the Doppler-derived effective orifice area. (c) AS grading as per *V*_max_ perspective. (d) AS grading as per mPG perspective. (e) Distribution of individual *V*_max_ values. (f) Distribution of individual mPG values. Complete sets of echocardiographic data at all four time points are given in Supporting Figure 1. Mean follow-up interval of complete sets as per *V*_max_ or AVA (*n* = 38): Postop.1: 11.3 ± 5.4 days; Postop.2: 15.2 ± 1.2 months; Last: 5.8 ± 0.5 years. Mean follow-up interval of complete sets as per mPG (*n* = 29): Postop.1: 10.8 ± 7.0 days; Postop.2: 15.7 ± 1.6 months; Last: 4.9 ± 0.5 years. The terminology “Preop.” refers to the assessment conducted just before MV surgery, “Postop.1” refers to the initial echocardiographic findings following MV surgery, “Postop.2” refers to results approximately one year after MV surgery, and “Last” designates the most recent echocardiographic assessment conducted prior to the AV intervention, aside from the one-year follow-up. In cases where the one-year results were the latest available, they were treated as the “Postop.1” assessment. Abbreviations: AS: aortic stenosis; AVA: aortic valvular orifice area; mPG: mean transaortic pressure gradient; *V*_max_: maximal transaortic velocity.

Postoperative changes in AS severity, as compared to preoperative grades based on parameters such as AV *V*_max_, AVA, and mPG, are depicted in Figure 2, respectively (complete sets of echocardiographic data at all four time points are given in Supporting Figure 1). Integrative assessment involves determining the severity of AS, particularly in cases of presumed severe AS, by considering not only the three classical parameters but also additional relevant factors. In cases of presumed high-gradient severe AS, careful evaluation of reversible causes that could lead to a significant increase in flow across the AV was emphasized. For patients with presumed LFLG severe AS despite preserved EF, the VTI ratio was incorporated into the assessment. Overall, the mean AV *V*_max_ changed from 2.2 ± 0.6 m/s to 2.4 ± 0.8 m/s and mPG changed from 11.1 ± 6.0 mmHg to 13.2 ± 8.2 mmHg. Six patients were classified as having severe AS. The serial-specific findings of the four true-severe AS patients (A, B, C, and D, 5.3%) and two LFLG pseudosevere AS patients (E and F) as per our assessments are summarized in Table 2 and Supporting Table 1, respectively.

#### 3.3.1. Patient A

A 33-year-old female with severe MS was referred to our institution for PMBV as she was planning for pregnancy. The procedure was successfully performed, and she had a stable delivery. After a 7-year gap in follow-up, she returned with dyspnea and underwent a redo PMBV, which yielded suboptimal results. Postprocedural echocardiography confirmed persistent severe MS with moderate MR (II/IV). Her AV parameters were *V*_max_ 4.5 m/s, peak/mean pressure gradient 80/45 mmHg, and mild AR (I/IV). Although the AVA was 1.3 cm^2^, inconsistent with typical high-gradient severe AS, there were no other identifiable flow-increasing conditions aside from MR (which would tend to reduce, rather than increase, transaortic gradients) and mild AR. Given the clinical context and hemodynamic profile, concomitant AV surgery was performed.

#### 3.3.2. Patient B

A 66-year-old female underwent mechanical MVR and tricuspid valvuloplasty for severe MS and moderate TR. At the time of surgery, her AV profile showed Vmax 3.0 m/s, mPG 19 mmHg, and AVA 1.0 cm^2^. Four years later, her *V*_max_ increased to 4.2 m/s, mPG to 38 mmHg, and AVA decreased to 0.9 cm^2^, meeting the criteria for high-gradient severe AS. However, as she remained asymptomatic, she was continued on regular follow-up.

#### 3.3.3. Patient C

A 60-year-old female underwent tissue MVR, tricuspid annuloplasty, and Maze operation for severe MS, TR, and AF at another hospital. Eighteen years later, she developed infective endocarditis requiring a redo DVR and repeat maze operation. Her last stable echocardiogram before surgery showed *V*_max_ 3.0 m/s, mPG 20 mmHg, AVA 1.0 cm^2^, SVi 24 mL/m^2^, EF 59%, and VTI ratio 0.25, consistent with paradoxical LFLG severe AS despite preserved LVEF.

#### 3.3.4. Patient D

A 43-year-old female with severe MS underwent PMBV at another hospital before transferring to our institution for follow-up. At 66 years, she underwent mechanical MVR, tricuspid valvuloplasty, and Maze operation. Five years postoperatively, her AV profile was *V*_max_ 3.5 m/s, mPG 30 mmHg, AVA 0.4 cm^2^, SVi 29 mL/m^2^, EF 59%, and VTI ratio 0.17, classifying her as paradoxical LFLG severe AS despite preserved LVEF. She is undergoing regular follow-up without any symptoms.

#### 3.3.5. Patient E (Summarized in Supporting Table 1)

A 51-year-old female underwent PMBV for severe MS. At that time, her AV profile was *V*_max_ 3.1 m/s, mPG 22 mmHg, AVA 1.1 cm^2^, and EF 66%. One year later, when she required MS surgery, her AV profile was *V*_max_ 3.1 m/s, mPG 20 mmHg, AVA 0.9 cm^2^, SVi 18, VTI ratio 0.31, and EF 62%, fulfilling paradoxical LFLG severe AS criteria. However, based on the VTI ratio and overall clinical assessment, true-severe AS was considered unlikely. Nonetheless, as the patient still had moderate AS, a DVR was performed alongside the MS surgery.

#### 3.3.6. Patient F (Summarized in Supporting Table 1)

A 49-year-old female underwent mechanical MVR for severe MS. At the time, her AV profile was *V*_max_ 2.6 m/s, mPG 15 mmHg, AVA 1.1 cm^2^, and EF 66%. Nine years later, her AV profile was *V*_max_ 3.8 m/s, mPG 29 mmHg, AVA 0.8 cm^2^, SVi 31, VTI ratio 0.33, and EF 64%, again suggesting paradoxical LFLG severe AS. Given the VTI ratio and clinical assessment, true-severe AS was considered unlikely, and she remained under regular follow-up.

Notably, four (Patients C, D, E, and F) of the six cases were classified as having paradoxical LFLG severe AS, meeting criteria of AVA < 1.0 cm^2^, *V*_max_ < 4.0 m/s, SVi < 35 mL/m^2^, and EF > 50%. None of the four patients underwent additional investigations such as dobutamine stress echocardiography or invasive valvular fractional flow reserve using a pressure wire. Only one patient underwent DVR as AV intervention for severe MS with LFLG true-severe AS. The other two patients (A and B) were deemed to have definitively severe AS and one of them underwent DVR as an AV intervention.

### 3.4. Progression of Rheumatic AR

**Figure 3:**
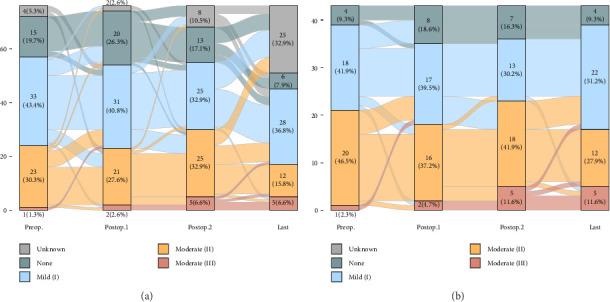
The changes in the grading of AR. No patient showed progression to severe AR during the follow-up. (a) Results with missing data across the four time points (*n* = 76). (b) Complete sets of echocardiographic data at all four time points (*n* = 43). Mean follow-up interval: Postop.1: 10.6 ± 4.8 days; Postop.2: 17.9 ± 2.4 months; Last: 6.3 ± 0.5 years. The terminology “Preop.” refers to the assessment conducted just before MV surgery, “Postop.1” refers to the initial echocardiographic findings following MV surgery, “Postop.2” refers to results approximately one year after MV surgery, and “Last” designates the most recent echocardiographic assessment conducted prior to the AV intervention, aside from the one-year follow-up. In cases where the one-year results were the latest available, they were treated as the “Postop.1” assessment.

There were no instances where postoperative AR severity progressed to severe; changes in severity grading are depicted in Figure 3.

Consequently, three out of the 76 patients underwent AVR, whose AV disease category was classified as severe AS.

In the group predominantly affected by severe MS for MV intervention, 16 showed deterioration of AS and only three demonstrated improvements. Among this group, 14 cases experienced an improvement in AR severity, while 15 exhibited an increased amount (Table 3).

### 3.5. Other Clinical Events

AF was newly diagnosed in 19 cases after MV intervention. Among them, 10 had undergone a Maze operation for AF previously, while the remaining nine cases developed de novo AF. Seven out of the nine cases with de novo AF were detected during the follow-up period after PMBV. There was one reported death among the 76 cases. Clinical events during follow-up are presented in Table 4.

## 4. Discussion

AS initiates damage in the left ventricle (LV) and may also lead to right ventricular injury, posing a risk of sudden cardiac death for the patient [13]. Approximately 60% of acute rheumatic fever patients go on to develop rheumatic heart disease, and while long-term or lifelong antibiotic therapy is recommended for secondary prevention in patients with established rheumatic valve disease, its implementation in routine clinical practice appears to be limited [14, 15]. Consequently, valve intervention at the appropriate disease stage remains as the cornerstone for managing rheumatic heart disease. Current guidelines recommend treating multiple valve disease according to the severity of the more affected valve. Valvular intervention may be warranted in cases of moderate AS accompanied by moderate AR, under specific circumstances [6, 16].

Diagnosing severe AS is a complex task and more so in the LFLG severe AS category. In our study, four cases (Patients C, D, E and F) are classified as having paradoxical LFLG severe AS despite normal EF category. This suggests a potential true-severe AS. While AVA, as an anatomical factor, indicates severe AS, the hemodynamic parameters, which lack sensitivity, do not fall within the high specificity range [17]. Calculating AVA using the continuity equation involves certain assumptions and is susceptible to intra- and interobserver variability. Factors contributing to inaccuracies in calculated AVA include controversies surrounding the measurement of left ventricular outflow tract (LVOT) diameter and the absence of adjustments for the elliptical shape of the LVOT [9, 18]. In addition, while averaging measured values in patients with AF is a widely adopted method, recent studies suggest a potential underestimation of AS severity with this practice [19]. One approach to address these challenges is to integrate the VTI ratio, which significantly enhances the sensitivity and specificity in grading severe AS [17, 20]. This integrated method proves practical in diagnosing cases of paradoxical LFLG severe AS, although the risk of measurement variability due to AF and irregular cycle length should be acknowledged. We addressed this limitation by averaging five consecutive beats in AF patients, following established protocols. With this approach, Patients E and F are classified as nonsevere AS cases. Even if Patient F does indeed have true-severe AS, the absence of related symptoms or signs suggests the need for regular follow-ups to monitor for the onset of symptoms rather than an immediate valve intervention unless indicated otherwise [21, 22].

The timing of AV assessment after MV intervention is a crucial factor. Generally, LV function and remodeling recover within the first year after MV intervention, at which point the AV can be evaluated in a stable condition [23]. This trend was also evident in our study, as severe AS was rarely observed in immediate postoperative TTE but most commonly identified at the 1-year follow-up. Beyond this point, further progression was uncommon, with minimal changes in AS severity seen in the final echocardiographic data. This suggests that the unveiling of severe AS at 1 year postoperatively likely results from the resolution of altered valve interactions following MV intervention, rather than true disease progression.

Invasive methods offer an alternative means to overcome the limitations of transthoracic echocardiography in grading AS severity. However, they present technical challenges and rely on a multitude of assumptions [24, 25].

The optimal timing for valve intervention in the paradoxical LFLG severe AS remains unclear. Proper management may involve addressing the potential impact of concurrent heart failure with preserved EF by treating hypertension and carefully monitoring the detrimental effects of AS on LV filling pressure, rather than focusing solely on AVA. Until further elucidation, general medical therapy for heart failure appears to suffice [25].

Regarding perioperative risk, it is inappropriate to directly compare MVR alone and DVR, given their distinct patient baseline comorbidities, which can vary from center to center. However, when examining mortality rates, DVR appears to have an overall 5-year mortality ranging from 5% to 20%, while MVR with or without tricuspid valvuloplasty demonstrates a relatively lower value [26–30].

As observed in this study, patients with rheumatic cardiac valve disease tend to be younger than those with degenerative cardiac valve disease and may opt for mechanical prosthetic valves to avoid the need for repeat replacement. Patients with dual mechanical valves face a higher risk of thrombosis and are recommended to maintain a higher target international normalized ratio [16].

There is no established lower threshold of mPG for mild AS. In our study, the same population was categorized into both the mild AS and no AS groups when sorted according to AV Vmax and AVA criteria. Consequently, an mPG of 14 mmHg was proposed as the lower limit cutoff for mild AS.

Among 18 patients whose preoperative assessment showed mild AS, five progressed to moderate AS, while one deteriorated to severe AS after four years of follow-up. Three out of nine patients who were assessed to have moderate AS at preoperative evaluation showed progression to severe AS after the median follow-up period of seven years (mean nine years). It is poorly understood why the patient with mild AS showed even more rapid progression to paradoxical LFLG severe AS than the moderate AS patients.

Looking at the changes in AS and AR severity in Table 4, aortic stenosis appears more likely to progress following the resolution of MS, whereas changes in AR are more variable. These findings likely reflect hemodynamic alterations rather than true structural progression or regression of the valvular lesions. It is currently understood that MR may underestimate the severity of AS, and in our study, AS and AR exhibited significant deterioration after MVR for severe MR in 25%. However, due to the small sample size in the predominantly severe MR group (four) and concurrent severe MS and MR group (only one), we cannot confidently speculate on the effect of resolving lesion interaction.

LVEF and LVWT significantly increased over time, while tissue Doppler early diastolic velocity (*T*_*E*−*e*′_) and the isovolumetric relaxation time (IVRT)/*T*_*E*−*e*′_ ratio significantly decreased. (Supporting Figure 2).

Overall, 3.4–10.3% of patients with rheumatic involvement at AV develop severe AS with a substantial portion in the form of paradoxical LFLG severe AS, and few of them exhibit severe AR during the median follow-up period of five to six years after MV intervention.

Our study has several limitations that need to be acknowledged. First, it is retrospective and observational in nature, which may introduce inherent biases. In addition, the presence of a substantial amount of missing data and a relatively short median follow-up period are important considerations. Focusing on patients with follow-up periods of over a year introduces potential survival bias, which should be taken into account when interpreting the results. Moreover, our study design does not allow us to make definitive conclusions about whether delaying AV intervention leads to improved clinical outcomes, especially in concerned patient groups. Identifying the specific factors contributing to patients falling into the paradoxical severe AS category and assessing whether patients undergoing MVR with rheumatic AV involvement are more susceptible to paradoxical severe AS were challenging. A larger sample size and further investigation may shed light on these aspects in future studies. The absence of long-term secondary prophylaxis with antirheumatic antibiotics represents another limitation of our study. This was primarily due to the low number of patients who started regular follow-ups before the age of 40 and the significant pain associated with benzathine penicillin G injections, even when mixed with lidocaine. Paradoxically, however, this lack of prophylaxis enabled us to more accurately demonstrate the natural progression of rheumatic heart disease.

## 5. Conclusion

In conclusion, only a small portion of rheumatic AV involvement progresses to severe AS after MV intervention, and performing MV intervention for severe MS or MR in patients with concurrent mild or moderate AS or AR due to rheumatic changes is considered safe.

## Figures and Tables

**Table 1:** Baseline characteristics (*n* = 76).

Age (years old)	56.5 ± 9.6
Female	53 (69.7)
Race	
Asian	76 (100)
Weight (kg)	57.6 ± 8.9
Height (cm)	158.6 ± 8.6
BMI	22.8 ± 2.9
Hypertension	5 (6.6)
Diabetes mellitus	8 (10.5)
Dyslipidemia	2 (2.6)
CVA	15 (19.7)
AF	60 (78.9)
Congenital heart disease	1 (1.3)
Long-term antirheumatic antibiotics	0 (0)
Indication for MV interventions	
Predominantly severe MS	71 (93.4%)
Predominantly severe MR	4 (5.3%)
Severe MS + severe MR	1 (1.3%)
AS severity grades	
Prestenotic rheumatic changes	42 (55.3)
Mild	18 (23.7)
Moderate	9 (11.8)
Severe	0 (0.0)
Unknown	7 (9.2)
EF (%)	56.6 ± 8.9
55∼	49 (64.5)
46–54	16 (21.1)
36–45	7 (9.2)
0–35	1 (1.3)
Unknown	3 (3.9)
MV intervention	
Mechanical MV replacement	54 (71.1)
Bioprosthetic MV replacement	9 (11.8)
MV ringed annuloplasty	1 (1.3)
Percutaneous mitral balloon valvuloplasty	12 (15.8)
Concurrent AF Op.	58 (76.3)
Concurrent TV Op.	26 (34.2)
Concurrent CABG Op.	1 (1.3)

Abbreviations: AF, atrial fibrillation; BMI, body mass index; CABG, coronary artery bypass graft; CVA, cerebrovascular accident; EF, ejection fraction; MR, mitral regurgitation; MS, mitral stenosis; MV, mitral valve; TV, tricuspid valve.

**Table 2:** Clinical information about AV of the four patients who progressed to severe aortic stenosis.

	Patient A	Patient B	Patient C	Patient D
Age at 1^st^ MV intervention	33	66	60	43
Sex	Female	Female	Female	Female
Follow-up (years)	7	4	18	28
AV profile on the preop. exam	^∗^Moderate AS	Moderate AS moderate AR (II/IV)	^∗^Mild AS	^∗^Missing
Valve intervention	PMBV for severe MS	Mechanical MVR + tricuspid valvuloplasty for severe MS and moderate TR	^∗^Tissue MVR + tricuspid annuloplasty + maze for severe MS and TR	PMBV mechanical MVR, tricuspid valvuloplasty, and maze operation for severe MS and severe TR
AV profile on the last exam	High-gradient severe AS Mild AR (I/IV)	High-gradient severe AS moderate AR (II/IV)	Paradoxical LFLG severe AS (VTI_LVOT_/VTI_AV_ = 0.25) Mild AR (I/IV)	Paradoxical LFLG severe AS (VTI_LVOT_/VTI_AV_ = 0.17) Mild AR (I/IV)
Additional valve intervention	DVR for severe MS and severe AS	No	DVR + maze for infective endocarditis	No

*Note:* As Patients A, C, and D underwent initial MV intervention at other institutions, much data are lacking. Patient C underwent DVR with maze operation not only because AS was severe but also because both MV and AV were involved with infective endocarditis. Patient D seems to have true-severe AS when the dimensionless parameter was integrated to assess the AS severity. Patients B and D are being followed with medical therapy including diuretics and show no limitation on daily activities. The term “preop.” refers to the assessment conducted just before MV surgery, and “last” refers to the most recent echocardiographic assessment conducted prior to the AV intervention, aside from the one-year follow-up.

Abbreviations: AR, aortic regurgitation; AS, aortic stenosis; AV, aortic valve; DVR, dual valve replacement (mitral and aortic valve replacement); LFLG, Low-flow low-gradient; LVOT, left ventricular outflow tract; MR, mitral regurgitation; MS, mitral stenosis; MV, mitral valve; MVR, mitral valve replacement; op., operation; PMBV, percutaneous mitral balloon valvuloplasty; TTE, transthoracic echocardiography; VTI, velocity-time integral.

^∗^Incomplete or missed data (data obtained from other hospitals).

**Table 3:** Postoperative changes in the severity grading of AS and AR.

Surgical indication	AS				AR			
	Improved	Maintained	Worsened	Missing	Improved	Maintained	Worsened	Missing
Predominantly severe MS (*n* = 71)	3	49	16	3	14	39	15	3
Predominantly severe MR	0	3	1	0	0	3	1	0
Severe MS + severe MR (*n* = 1)	1	0	0	0	0	1	0	0

*Note:* Severity assessments using echocardiography, at least one year after mitral valve surgery, compared to preoperative evaluations are shown. Among the three patients with paradoxical low-flow, low-gradient severe aortic stenosis despite normal left ventricular ejection fraction, only one patient was classified as having true-severe aortic stenosis according to our assessment. The remaining two patients were classified as having moderate aortic stenosis. Parenthesis presents the number of patients.

Abbreviations: AR, aortic regurgitation; AS, aortic stenosis; MR, mitral regurgitation; MS, mitral stenosis.

**Table 4:** Clinical events during follow-ups.

Clinical events	Cases
Death	1
Cardiovascular death	0
Sepsis	^∗^1
Heart failure hospitalization	3
Unstable angina	1
Infective endocarditis	2
Structural valve deterioration	^†^1
Nonstructural valve deterioration	0
Valve thrombosis	0
New AF	19
Recurrence after Maze op.	10
De novo	9
CIED	2
Permanent pacemaker	1
Implantable cardiac defibrillator	1

*Note:* Clinical events occurred during the follow-ups after the MV intervention was presented.

Abbreviations: AF, atrial fibrillation; CIED, cardiac implantable electronic device.

^∗^Patient died of infective endocarditis. Surgery was not performed.

^†^Bioprosthetic valve tear.

## Data Availability

The data used to support the findings of this study are available from the corresponding author upon request.

## Nomenclature

AF: Atrial fibrillation
AR: Aortic regurgitation
AS: Aortic stenosis
AV: Aortic valve
AVA: Aortic valvular orifice area
AVR: Aortic valve replacement
DVR: Double valve replacement
EF: Ejection fraction
IVRT: Isovolumetric relaxation time
LFLG: Low-flow, low-gradient
LVOT: Left ventricular outflow tract
mPG: Mean transaortic pressure gradient
MR: Mitral regurgitation
MS: Mitral stenosis
MV: Mitral valve
MVR: Mitral valve replacement
PMBV: Percutaneous mitral balloon valvuloplasty
TV: Tricuspid valve
*V*_max_: Maximal transaortic velocity

## Supporting Information

Supporting Table 1. Clinical Information about two patients with LFLG severe aortic stenosis despite preserved left ventricular ejection fraction and deemed to have pseudosevere AS. Patient E underwent DVR with tricuspid annuloplasty for severe MS accompanied by paradoxical LFLG severe AS. Patient F was deemed to have high-gradient severe AS based on the echocardiographic assessment at one year after 1^st^ MV intervention and as the last assessment was made in the context of infective endocarditis. Abbreviations: AR, aortic regurgitation; AS, aortic stenosis; AV, aortic valve; DVR, dual valve replacement (mitral and aortic valve replacement); LFLG, low-flow low-gradient; LVOT, left ventricular outflow tract; MR, mitral regurgitation; MS, mitral stenosis; MV, mitral valve; MVR, mitral valve replacement; op, operation; PMBV, percutaneous mitral balloon valvuloplasty; TTE, transthoracic echocardiography; VTI, velocity-time integral. ^∗^Further echo data became irrelevant as the patient underwent DVR with tricuspid annuloplasty after follow-up echocardiography at one year after the PMBV.

Supporting Figure 1. The changes in the grading of AS. The changes in the grading of aortic stenosis of patients with complete sets of echocardiographic data as per four standards are presented. (A) AS grading as per integrative assessment, incorporating the velocity-time integral ratio to classify low-flow, low-gradient severe AS despite preserved left ventricular ejection fraction. (B) AS grading as per AVA perspective, where AVA was calculated using the continuity equation as the Doppler-derived effective orifice area. (C) AS grading as per *V*_max_ perspective. (D) AS grading as per mPG perspective. AS: aortic stenosis; AVA: aortic valvular orifice area; mPG: mean transaortic pressure gradient; *V*_max_: maximal transaortic velocity.

Supporting Figure 2. Postperative changes of several indices. EF was obtained using Simpson's method. LVWT was measured at the 2-dimensional parasternal long-axis view. Although accurate measurements for *T*_*E*−*e*′_ require dual Doppler echocardiography, the assessment in this study was made retrospectively using separate views. The terminology “Preop.” refers to the assessment conducted just before MV surgery, “Postop.1” refers to the initial echocardiographic findings following MV surgery, “Postop.2” refers to results approximately one year after MV surgery, and “Last” designates the most recent echocardiographic assessment conducted aside from the one-year follow-up. EF: ejection fraction; IVRT: isovolumetric relaxation time; LV: left ventricle;  *T*_*E*−*e*′_: tissue Doppler early diastolic velocity; WT: wall thickness.
