# Supplementary material for: Natural Progression of Rheumatic Aortic Valve Disease Following Mitral Valve Intervention: A 16-Year Single-Center Experience
Source: Cardiol Res Pract. 2025 Jun 17;2025:6689214. doi: 10.1155/crp/6689214 (PMC12187430; doi:10.1155/crp/6689214)
Supplement: Supporting Information — Additional supporting information can be found online in the Supporting Information section. [file 6689214.f1.docx]

SUPPLEMENTARY MATERIALS

Supplemental Table 1. Clinical Information about two patients with LFLG severe aortic stenosis despite preserved left ventricular ejection fraction and deemed to have pseudo-severe AS

|  | Patient E | Patient F |
| --- | --- | --- |
| Age at 1^st^ MV intervention | 51 | 49 |
| Sex | Female | Female |
| Follow-up (years) | 3 | 9 |
| AV profile on the pre-op. exam | Moderate AS  Mild AR(I/IV) | Mild AS  Moderate AR(II/IV) |
| Valve  Intervention | PMBV for severe MS | Mechanical MVR for severe MS |
| AV profile on the last exam | * No | Paradoxical LFLG Severe AS (VTI_LVOT_/VTI_AV_ = 0.32)  Moderate AR(III/IV) |
| Additional  valve intervention  including AV | DVR + Tricuspid annuloplasty for severe MS accompanied by paradoxical LFLG severe AS | No |

Patient E underwent DVR with tricuspid annuloplasty for severe MS accompanied by paradoxical LFLG severe AS

Patient F was deemed to have high gradient severe AS based on the echocardiographic assessment at one year after 1^st^ MV intervention and as the last assessment was made in the context of infective endocarditis.

Abbreviations: AR, aortic regurgitation; AS, aortic stenosis; AV, aortic valve; DVR, dual valve replacement (mitral and aortic valve replacement); LFLG, Low flow low gradient; LVOT, left ventricular outflow tract; MR, mitral regurgitation; MS, mitral stenosis; MV, mitral valve; MVR, mitral valve replacement; Op., Operation; PMBV, percutaneous mitral balloon valvuloplasty; TTE, Transthoracic echocardiography; VTI, velocity-time integral.

* Further echo data became irrelevant as the patient underwent DVR with tricuspid annuloplasty after follow-up echocardiography at one year after the PMBV

Supplementary Figure 1. The changes in the grading of AS


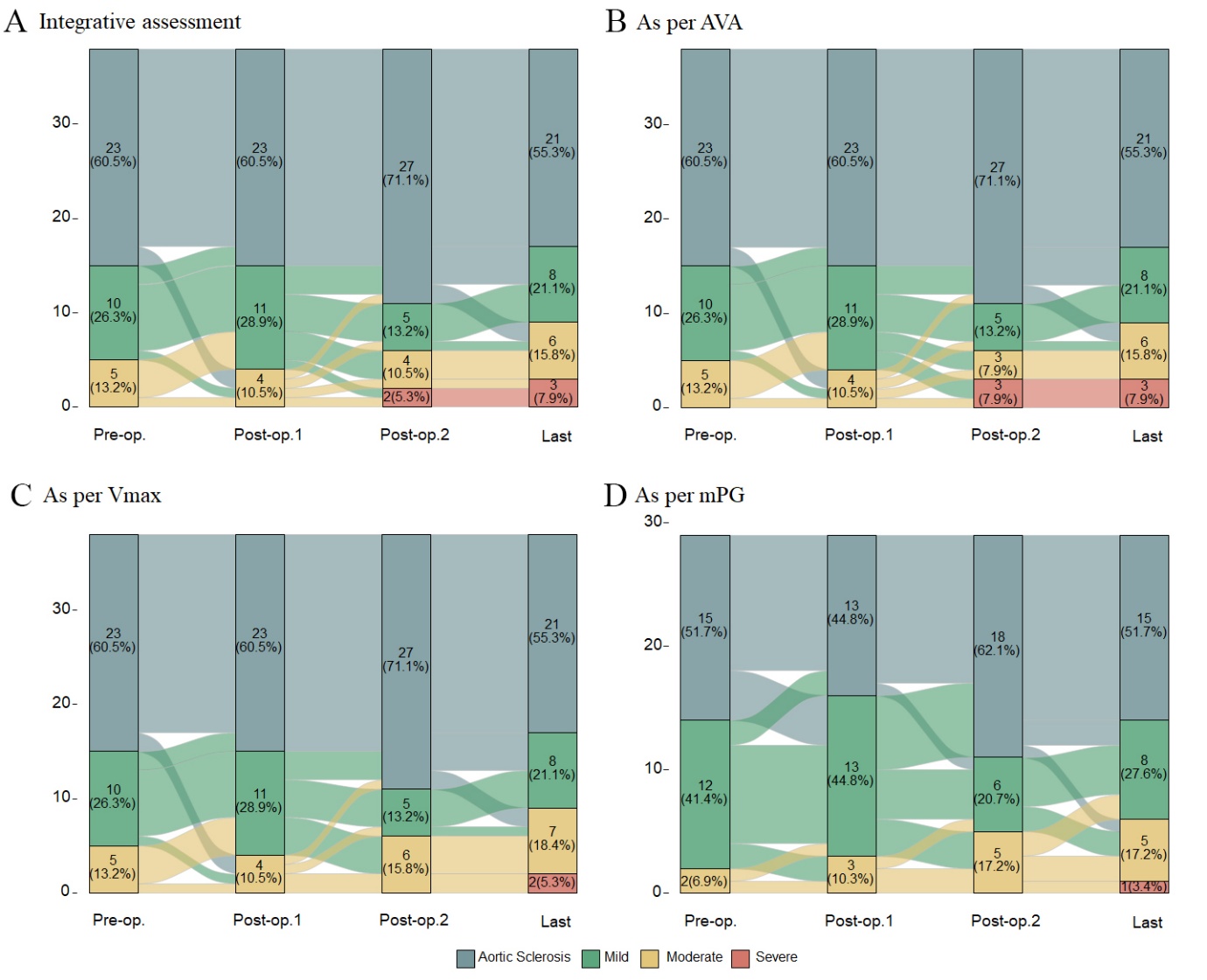


The changes in the grading of aortic stenosis of patients with complete sets of echocardiographic data as per four standards are presented.

(A) AS grading as per integrative assessment, incorporating the velocity time integral ratio to classify low-flow, low-gradient severe AS despite preserved left ventricular ejection fraction.; (B) AS grading as per AVA perspective, where AVA was calculated using the continuity equation as the Doppler-derived effective orifice area; (C) AS grading as per Vmax perspective; (D) AS grading as per mPG perspective.

AS: Aortic stenosis, AVA: Aortic valvular orifice area, mPG: Mean transaortic pressure gradient, Vmax: maximal transaortic velocity

Supplementary Figure 2. Post-operative changes of several indices.


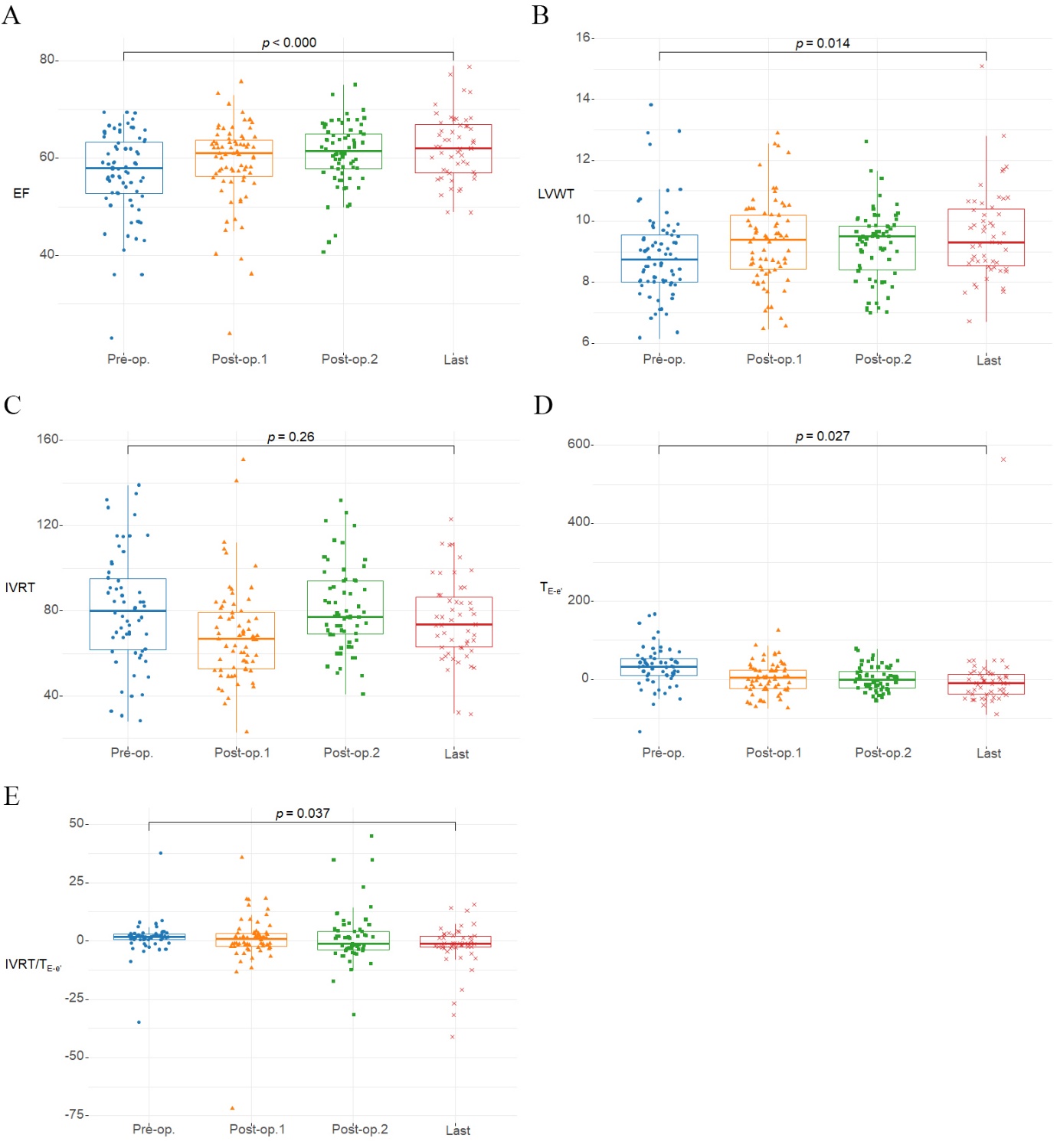


EF was obtained using Simpson’s method. LVWT was measured at the 2-dimensional parasternal long axis view.

Although accurate measurement for T_E-e'_ requires dual Doppler echocardiography, the assessment in this study was made retrospectively using separate views.

The terminology "Pre-op." refers to the assessment conducted just before MV surgery, "Post-op.1" refers to the initial echocardiographic findings following MV surgery, "Post-op.2" refers to results approximately one year after MV surgery, and "last" designates the most recent echocardiographic assessment conducted aside from the one-year follow-up.

EF: ejection fraction, IVRT: isovolumic relaxation time, LV: left ventricle, T_E-e'_: tissue Doppler early diastolic velocity, WT: wall thickness
